# Intra-operative ultrasound: does it improve the results of percutaneous repair of acute Achilles tendon rupture?

**DOI:** 10.1007/s00068-022-01926-x

**Published:** 2022-03-11

**Authors:** Ahmed Mohamed Samy

**Affiliations:** Orthopedic Department, Tanta University, Tanta, Egypt

**Keywords:** Ultrasound, Percutaneous repair, Achilles tendon, Sural nerve

## Abstract

**Purpose:**

Percutaneous repair is a good option for acute Achilles tendon rupture. Although it overcomes the complications of open technique, it carries the risk of sural nerve injury and inadequate repair. In this study, we explore if the use of intra-operative ultrasound with percutaneous technique has any advantageous effect on final results of repair.

**Methods:**

This is a prospective randomized study done between May 2014 and December 2020. It included 91 patients with complete acute Achilles tendon rupture distributed in 2 groups with homogenous clinical and demographic data. Group A (*n* = 47) included those managed by percutaneous repair with assistant of an intra-operative ultrasound. Group B (*n* = 44) included those done without the assistant of ultrasound. Post-operative evaluation was done clinically by the American Orthopedic Foot and Ankle Society score, calf muscle circumference and single heel rise test and radiologically by Magnetic Resonance Image.

**Results:**

Patients of both groups reported continuous improvement of the American Orthopedic Foot and Ankle Society score with time. However, patient of group A recorded better functional outcome score at 3 months postoperatively. We recorded longer operative time in group A than those in group B. Continuous improvement of maximum calf circumference was observed in both groups. Satisfactory healing was noticed to happen faster in patients of group A than those of group B. We recorded two cases of re-rupture and two cases of sural nerve injury in group B with no reported complication in group A.

**Conclusion:**

The use of an intra-operative ultrasound with percutaneous repair of acute rupture of Achilles tendon can improve the quality of repair as evidenced by quicker satisfactory healing and earlier regain of activity. Also, it can help in proper localization of sural nerve in relation to lateral edge of Achilles tendon.

**Trial registration:**

Clinical Trials.gov Identifier: NCT04935281.

## Introduction

Rupture of Achilles tendon is a common tendon injury especially among men between 30 and 40 years old. It may occur due to direct trauma or sudden plantar flexion of the foot especially with ankle pronation. The incidence increases with poor muscle condition, extreme physical exercise, prolonged intake of corticosteroids and patients with systemic disease, e.g. autoimmune diseases and diabetes mellitus [1]. Till now, there is a great debate about the ideal way to manage this problem. Many reports recorded good results with non-operative management [2, 3]. However, due to high rate of re-rupture and delayed recovery, many surgeons prefer the surgical repair [3]. Open technique was the traditional way of repair for many decades [4]. Because of the relatively high incidence of wound complications and adhesions, percutaneous and minimally invasive surgical repairs were introduced to overcome these challenging complications [5].

The percutaneous techniques have gained popularity over years since their appearance by Ma and Griffith in 1977 [6]. The main drawbacks of this technique are the sural nerve injury and tendons re-rupture. Later on, mini-open techniques gained interest with use of variable devices to overcome these problems. Among these devices, Dresden instrument [7] and Achillon jig [8] were the most common. They depend on sub-fascial technique of applying the suture in order to avoid nerve injury. However, the need for special instruments, long learning curve needed and high costs of these devices are the main limitations of mini-open techniques [3].

This study was conducted in order to compare the results of percutaneous repair of acute Achilles tendon rupture with or without assistance of an intra-operative ultrasound. We assumed that the use of an intra-operative ultrasound can improve the technique of percutaneous repair and at the same time reduce the incidence of potential complications.

## Methods

This is a prospective randomized controlled study (level of evidence I) carried out between May 2014 and December 2020. It included 98 patients presented with acute rupture of Achilles tendon. All procedures were in accordance with the ethical standards of the local institutional committee on human experimentation and have been performed in accordance with the ethical standards as laid down in the 1964 Declaration of Helsinki and its later amendments.

Informed consents were taken from all patients. All patients subjected to a percutaneous repair of Achilles tendon with or without assistant of an intra-operative ultrasound. The patients were randomly distributed by closed envelop technique (49 in each group). Group A included those managed with the assistant of an intra-operative ultrasound. Group B included those done without ultrasound assistant.

Our inclusion criteria were: age between 18 and 50 years with acute (≤ 2 weeks after trauma) closed complete injury of Achilles tendon in zone 2 (the area between 3 and 6 cm from the insertion) according to Langergran and Lindholm [9]. Exclusion criteria were: incomplete or recurrent injury, ipsilateral ankle or foot fracture, previous history of local corticosteroids injection, patients with neurovascular problem (e.g. diabetic, autoimmune …etc.), smoking, and alcoholics.

During follow-up period, seven patients were further excluded from the study; three patients did not complete the follow-up and missed 2 months postoperatively and four patients have additional orthopedic problems in the same limb within 8 months postoperatively. Accordingly, our materials included 91 patients, 47 in group A and 44 in group B. The patients’ demographic and clinical data are summarized in Table 1.

**Table 1:** Patients’ demographics and clinical characteristics

Demographic and clinical data	Group A (*n* = 47)	Group B (*n* = 44)	Total (*n* = 91)	*p* value
Age (years): mean ± SD (range)	30.71 ± 5.15 (21–49)	31.71 ± 8.44 (19–50)	31.15 ± 4.19 (19–50)	*T*: 0.468
Gender (male: female)	35:12	37:7	72:19	*X*^*2*^: 0.925
BMI (kg/m^2^): mean ± SD (range)	30.65 ± 1.96 (22–35)	31.25 ± 2.82 (21–34)	30.94 ± 2.17 (21–35)	*T*: 0.484
Cause of injury (*n* of cases)				
Sport’s activity	25	28	53	*X*^*2*^: 0.624
Falling downstairs	15	11	26	
Direct trauma	7	5	12	
Injured side (Rt:Lt)	22:25	24:20	46:45	*X*^*2*^: *0.357*
Time lag before surgery (days): mean ± SD (range)	4.19 ± 1.51 (1–13)	4.31 ± 1.68 (3–14)	4.03 ± 1.27 (1–14)	*T*: 0.442

*BMI* body mass index, *T* paired *t* test, *X*^2^ chi-square test

The diagnosis established clinically by positive Thompson test, loss of active planter flexion, and a palpable gap. All patients had undergone MRI preoperatively to confirm the injury and measure the length of the distal stump.

## Surgical technique

A preoperative prophylactic dose of antibiotic was given intravenously in the form of 2gm of cephalosporin 1 h before operation.

Under general or regional anesthesia, the patient laid in prone position without a tourniquet and both feet out of the table for easily mobilization of the ankle joint. In group A, an intra-operative ultrasound was done by a radiologist before the repair for identification the course of sural nerve and outline the medial and lateral edges of torn tendon. Actually, it was difficult to visualize the sural nerve with ultrasound. Anatomically, the small saphenous vein is located on the medial side of the nerve and it was easier to visualize it than the nerve. Therefore, we were able to localize the course of the nerve in all cases of group A by identification the course of small saphenous vein [10].

Ultrasound was repeated after repair for confirmation of adequate contact of both stumps and satisfactory strength of the repair by visualization of the tendon with passive motion of the ankle. In case of insufficient repair and presence of any gap between ends, the sutures were withdrawn and redone again. In some cases, it was difficult to withdraw the sutures. Therefore, another reinforcement sutures were done by the same technique.

For patients in group B, the course of the sural nerve was determined according to the technique described by Blackmon et al. [11]. This technique depends on the leg length for location the point where the nerve crosses the lateral edge of the tendon [11]. The medial and lateral borders of both stumps were outlined by palpation with identification of the gap in-between.

**Fig. 1:**
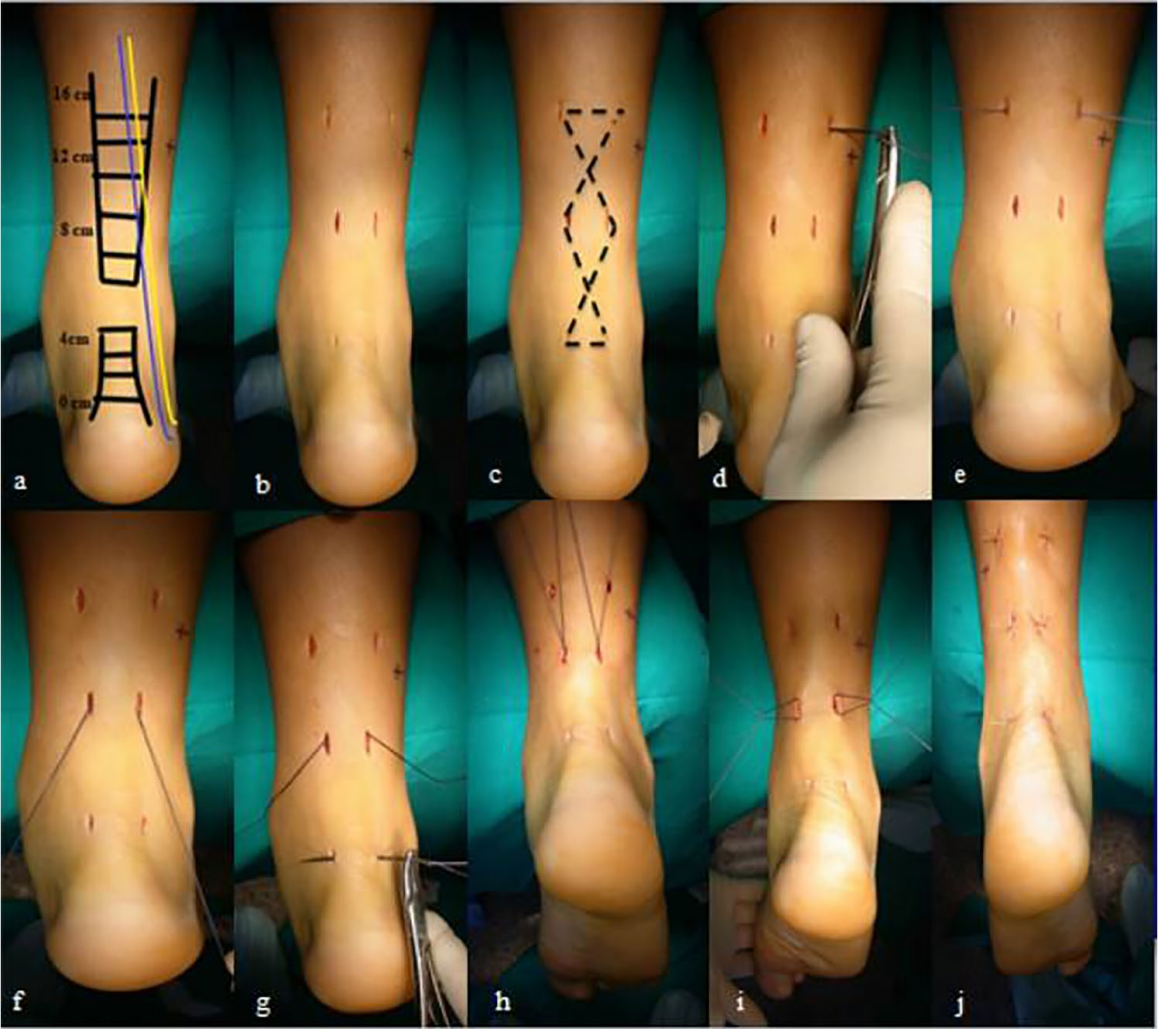
**a** Marking the course of sural nerve (yellow line) and small saphenous vein (blue line) in relation to Achilles tendon. **b** Stab incisions. **c**–**j** Surgical technique and advancement of the suture through both stumps

**Fig. 2:**
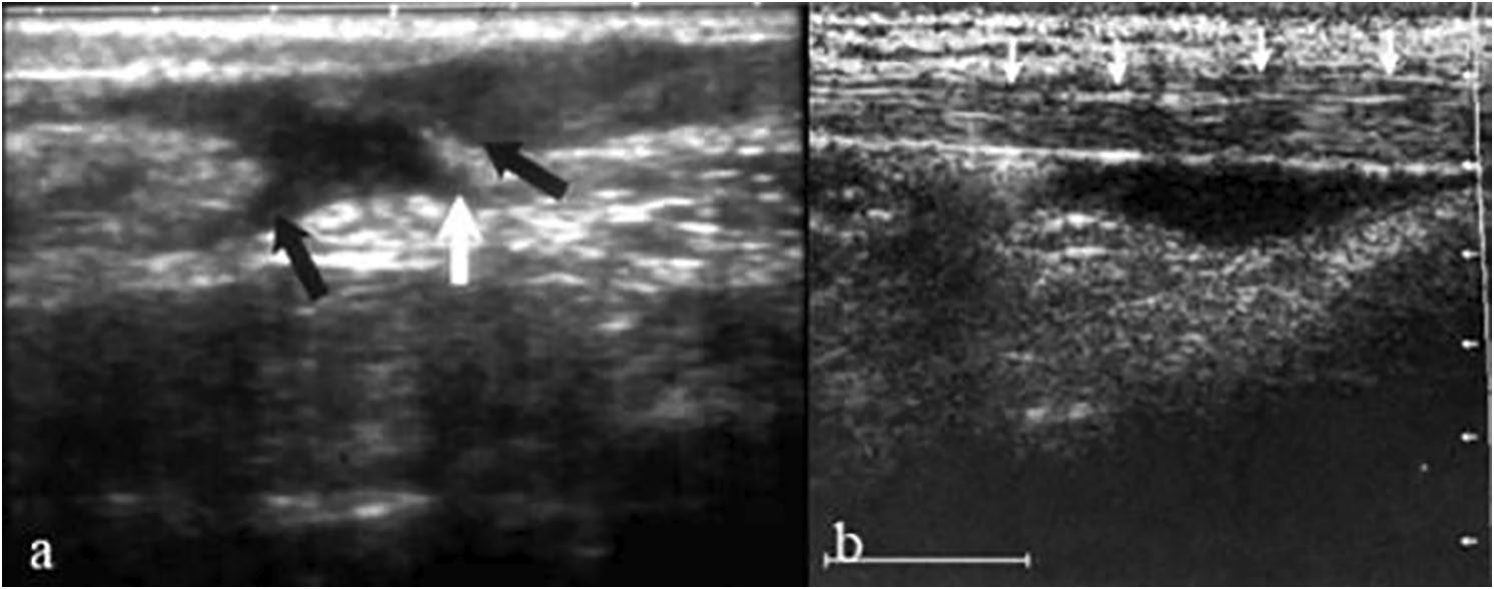
**a** Intra-operative ultrasound before repair (black arrows represent torn edges and white arrow represents sural nerve. **b** Intra-operative ultrasound after repair with good contact between torn edges

The technique of repair was standardized for all patients in both groups. We used the technique described by Maffulli et al. [12] with six stab incisions, one cm each. Four incisions were on medial and lateral edge of the proximal stump and the other two were around distal stump. We used for repair Ethibond size 5 with double large needles (Ethicon, Somerville, NJ) (Figs. 1, and 2).

After repair, passive motion of the ankle was done to ensure absence of any attachment with the skin and to confirm sufficiency of repair. Stab incisions closed with absorbable sutures. A below knee cast was applied in 30 degrees of plantar flexion.

## Post-operative regimen

A prophylactic low molecular weight heparin was given for 6 weeks postoperatively. First cast continued for 2 weeks. After that, the cast was removed and wound was inspected. Another below knee cast was applied with ankle in neutral position for additional 4 weeks.

During casting, active quadriceps exercises were encouraged. Active and passive exercises of the ankle were allowed after removal of the cast with partial weight bearing with 1.5 cm heel lift for additional 6 weeks.

**Fig. 3:**
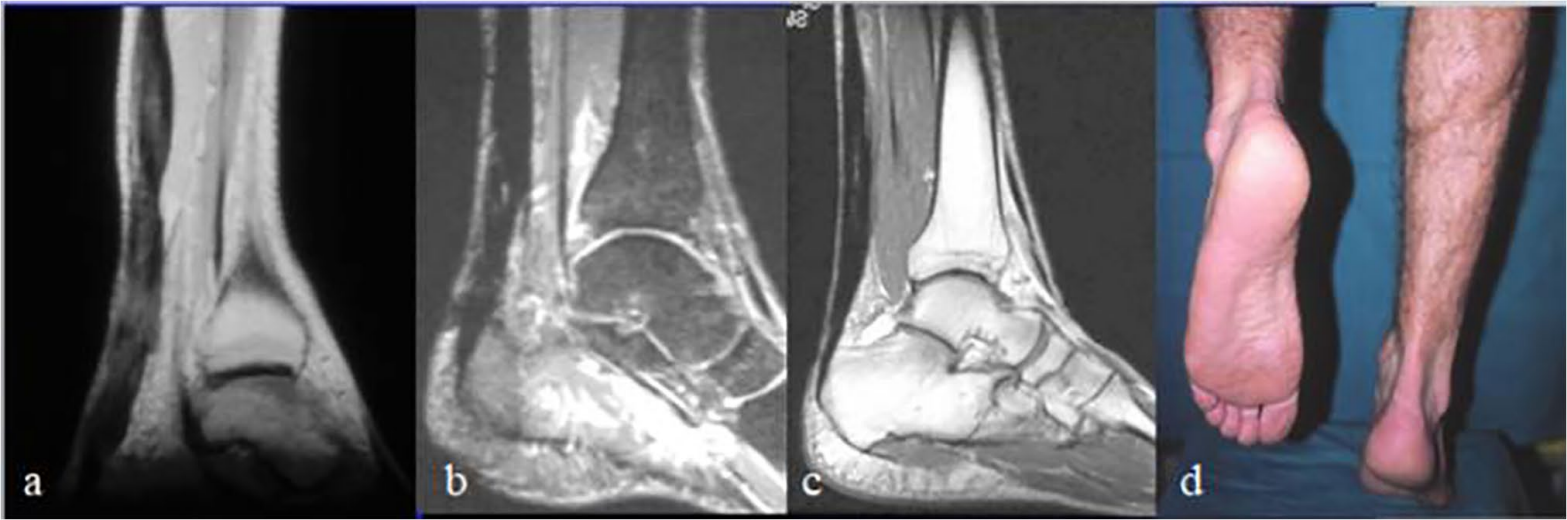
**a** Magnetic resonance imaging scan of male patients 34 years old 3 months postoperatively with satisfactory healing. **b** Magnetic resonance imaging scan 6 months postoperatively. **c** Magnetic resonance imaging scan at final follow-up. **d** Single heel rise 3 months postoperatively

MRI was done 3 months postoperatively to ensure good healing followed by full weight bearing. Sports activities were permitted according to adequacy of healing guided by clinical examination and MRI. Adequacy of healing can be assessed on the sagittal images of the MRI. The gap between torn ends may persist up to 2 months after repair with ill-defined T2 weighted signal. After that, the gap gradually decreased and replaced by fibrous tissue with hypo intense weighted image. The healing process starts from the peripheral of the tendon to the center. Good and satisfactory healing can be evaluated by disappearance of the gap and appearance of hypo intense fibrous tissue in T1and T2 across the gap in addition to decrease in the size of the tendon [13] (Fig. 3).

Clinical evaluation was done immediately after cast removal then at monthly intervals during first 6 months. Then, at 3-month intervals till the end of the follow-up.

Clinical evaluation included: measurement of calf muscle circumference (expressed as the difference between healthy and injured side in millimeters) [14], single heel rise [15], ankle range of motion and compared to the healthy side. Functional evaluation was done by American Orthopedic Foot and Ankle Society score (AOFAS) [16].

## Statistical analysis

Analysis was done with SPSS 16.0 (IBM, USA). The comparison between both groups were done by the chi-square test for categorical value and presented as a count. Student *t* test was used for quantitative value and presented as a mean ± standard deviation. *p* value less than 0.05 was considered as a significant value.

## Results

Both groups were comparable as regards age, gender, BMI, cause of injury, time lag before surgery, period of hospitalization and follow-up period (Tables 1 and 2). The use of intra-operative ultrasound seems to have more time consumption. We recorded longer operative times for patients in group A in comparison to those in group B and this was statistically significant (*p* = 0.036). However, patient of group A recorded better functional outcome score at 3 months postoperatively as measured by AOFAS and this was statistically significant (*p* = 0.042). Later on, non-significant difference was recorded between groups.

**Table 2:** Post-operative clinical and radiological results

Studied variable	Group A Mean ± SD	Group B Mean ± SD	*p* value
Operative time (minutes)	47.22 ± 7.92	28.25 ± 4.16	0.036
Time of hospitalization (days)	1.80 ± 0.66	1.51 ± 0.30	0.652
Follow-up period (months)	51.38 ± 3.12	47.52 ± 2.70	0.318
Post-operative AOFAS			
3 months	90.71 ± 3.61	81.42 ± 6.15	0.042
6 months	94.28 ± 4.30	92.11 ± 5.17	0.725
Final follow-up	98.11 ± 1.72	97.68 ± 2.10	0.533
Difference in MCC (mms)			
3 months	30.35 ± 1.82	34.72 ± 1.20	0.831
6 months	15.44 ± 1.07	17.57 ± 1.82	0.629
Final follow-up	1.59 ± 0.72	1.61 ± 0.32	0.527
Satisfactory healing in MRI (weeks)	12.68 ± 2.19	21.26 ± 1.19	0.014
Time to work (weeks)	11.37 ± 3.19	11.68 ± 4.19	0.516
Time to sport (weeks)	15.18 ± 3.11	23.48 ± 2.58	0.042*

*AOFAS* American Orthopedic Foot and Ankle Society score, *MCC* maximum calf circumference, *MRI* magnetic resonance imaging

*Significant at *p* value < 0.05 using paired *t* test

MRI follow-up at 3 months postoperatively showed satisfactory healing in 41 patients (87.23%) of group A and in only 29 patients (65.91%) of group B and this was statistically significant (*p* = 0.037). Satisfactory healing was noticed to happen earlier in patients of group A than those of group B and this was statistically significant (*p* = 0.014).

We measured the improvement in calf muscle circumference as a measure of functional recovery. Continuous improvement of maximum calf circumference was observed in both groups. The improvement was to some extent superior in group A in comparison to group B but this was statistically insignificant (*p* = 0.527). At the final follow-up, the difference in circumference between injured and non-injured limb was irrelevant. Patients of group A returned to sports activity earlier than those of group B and this was statistically significant (*p* = 0.042) (Table 2).

Intra-operatively, the repair was repeated in three cases of group A and one case of group B due to insufficient contact between torn ends. As regards group A, Insufficient repair was discovered clinically by persistent of the gap and confirmed by ultrasound. For group B, the gap was discovered by clinical palpation. This was statistically insignificant (*p* = 0.730).

We did not record any incidence of D.V.T. (deep venous thrombosis), infection nor skin problems. As regards re-rupture, we did not record any case in group A. However, two cases of re-rupture were reported in group B. One case occurred after minor trauma 10 weeks postoperatively and other had spontaneous rupture 12 weeks postoperatively. Cases of re-rupture were managed by open technique with augmentation. At the final follow-up, all cases in both groups except cases of re-rupture were able to do single heel rise sufficiently.

We recorded two cases of hyperesthesia in area supplied by sural nerve. Both patients were in group B and resolved completely with medical treatment.

## Discussion

The treatment of acute rupture of Achilles tendon represents a great challenge with many debatable modalities of management. The risks and complications of every method either conservative or surgical made a burden on the surgeon to choose which method is more suitable for the patient. The high incidence of re-rupture with conservative treatment was a main cause that many surgeons preferred surgical interference. However, open techniques carry the risk of many complications such as infection and adhesion. On the other side, the percutaneous and min-invasive techniques have the hazard of unsatisfactory repair and sural nerve injury [17, 18].

Many trials were done to decrease the incidence of these complications. Different assistant devices were developed to overcome these drawbacks [7, 8]. Indirect visualization of the torn tendon and sural nerve by intra-operative ultrasound and endoscope was another way to improve the technique of percutaneous repair [19].

This study was done in order to explore if the use of an intra-operative ultrasound with percutaneous repair for acute rupture of Achilles tendon can improve the technique and reduce the incidence of complications.

In our study, patients of both groups reported continuous improvement of AOFAS with time. This was in harmony with results recorded by Moller et al. [4] and Twaddle et al. [20]. However, patients done with assistance of ultrasound reported superior AOFAS in early follow-up and this was statistically significant (*p* = 0.042). Enhanced results in group A patients could be attributed to accurate needle pass and adequate grip in the tendon that allow sufficient strength of applied sutures and insure absence of any gap between proximal and distal stump. Our results could be clarified by the work of Soubreyrand et al. who reported satisfactory placement of suture in all cases done with intra-operative assistance of ultrasound. In contrast, only 55% of cases done without ultrasound were properly placed in the tendon [19].

Atrophy of calf muscle after Achilles tendon injury is a known squeal with conservative or operative treatment. Some studies documented that complete recovery may take up to 33 months after injury [21]. Others, concluded that complete recovery never be returned even over time [22, 23]. In contrast, at the end of follow-up, we observed patients of both groups gained nearly normal calf muscle circumference in comparison to uninjured limb with no significant difference between groups.

We did not record any significant effect of time lag before surgery on the functional outcomes. We claimed this to early interference in all cases within 2 weeks of injury as recommended by Carden et al. to avoid adhesion [24].

As regards time to return to work, no difference was reported between groups and it was nearly after 12 weeks. This was similar to previous reports which reported average time between 12 and 18 weeks [1, 25, 26]**.**

Data about the optimum time for return to sports activity are scarce [27]. In general, it is recommended to avoid sports up to 20 weeks after repair [28]. In our study, we depend on MRI follow-up to allow return to sports activity. Patients of group A recorded satisfactory healing in MRI earlier than those of group B and this was statistically significant (*p* = 0.014). So patients of group A returned earlier to sports (15.18 ± 3.11 weeks) than those of group B (23.48 ± 2.58 weeks) and this difference was significant (*p* = 0.042).

As regards, operative time, percutaneous repair guided with an intra-operative ultrasound was a time consuming procedure especially at the beginning of the study. Later on, with increased experience and learning curve it decreased dramatically. Therefore, patients of group A recorded longer operative time in comparison to those of group B and this was statistically significant (*p* = 0.036).

In our series, the only reported complications were re-rupture and sural nerve injury. The reported two cases of re-rupture occurred in group B with overall incidence 2.10%. No significant difference was identified between groups. This incidence was in harmony with many studies that reported a mean incidence 2.6% [2, 29, 30] and was better than recorded by Reito et al. and Rettig et al. with incidence 7.1% and 4.5%, respectively [31, 32]

The anatomical course of the sural nerve has many individual variations. There is a controversy about the exact location where the nerve crosses the lateral border of the tendon. The distance from tendon insertion ranged between 5.7 and 11.cm [33]. This variability made sural nerve injury one of the main drawbacks of percutaneous repair. Although, Ma and Griffith did not record any incidence of nerve injury in their original work [6]. Many studies documented this complication as a main problem of percutaneous technique with a mean incidence 7.29% [2, 29, 33]. However, Klein reported a higher rate of incidence of 13% [34]. Porter reported 27% incidence in his cadaveric study [35]. To overcome this problem, some advised the necessity of visualization of the nerve by extending the lateral stab incision [36], others recommended to avoid lateral stab incisions and used instead mid-line stab incisions [37]. The use of ultrasound reported a decrease in the incidence of sural nerve injury. Giannetti et al. have no case of nerve injury with the use an intra-operative ultrasound [38]. Pavic recommended a direct visualization by endoscope [39].

Some series reported a high rate of sural nerve injury with percutaneous technique up to 18% [36]. Haji reported 10.5% sural nerve injury [40].

In group B, we identify the anatomical course of sural nerve according to cadaveric study done by Blackmon et al. [11] who concluded a mean distance between the course of the nerve and lateral border of Achillis tendon as measured from its calcaneal insertion. In our series, we recorded only two cases (2.20%) of sural nerve injury. Our finding was similar to that obtained by Soubeyrand et al. [19] who used intra-operative ultrasound and superior to the results of Fortis et al. [41] who used endoscope with percutaneous repair and reported sural nerve incidence in 10% of cases. The two cases were reported in group B, but due to low incidence of this complication in our series we did not detect any significant difference between both groups.

Although we recorded better recovery, earlier return to activity and fewer incidences of complications, the main drawbacks of this technique is the need for experienced radiologist and more time consumption.

The strengths of this study are that it included a control group; both groups were comparable as regards demographic and clinical data, all surgeries were done by the same surgeon and relatively sufficient period of follow-up to detect deterioration or complications.

The main limitations of this study were missing data about the cost of surgery in each group, absence of data about a pre-injury sport performance to compare the postoperative activity level to the preoperative one including type of sports, frequency and performance. Also, we did not record the changes in the length of Achilles tendon after repair over the period of follow-up which has direct effect on overall tension of musculo-tendinous unit.

## Conclusion

The use of an intra-operative ultrasound with percutaneous repair of acute rupture of Achilles tendon can improve the quality of repair as evidenced by earlier satisfactory healing and regain of activity. However, it is to be viewed critically in terms of implementation in clinical practice due to significantly longer operation time and the need for an experienced radiologist. Although we did not detect any significant difference between both groups as regards sural nerve affection, ultrasound can help to some extent in proper localization of sural nerve in relation to lateral edge of Achilles tendon.

## Data Availability

The data sets generated during and analyzed during current study are available from the corresponding author on reasonable request.
